# Breeding for Anthracnose Disease Resistance in Chili: Progress and Prospects

**DOI:** 10.3390/ijms19103122

**Published:** 2018-10-11

**Authors:** Raihana Ridzuan, Mohd Y. Rafii, Siti Izera Ismail, Martini Mohammad Yusoff, Gous Miah, Magaji Usman

**Affiliations:** 1 Laboratory of Climate-Smart Food Crop Production, Institute of Tropical Agriculture and Food Security, Universiti Putra Malaysia, Serdang 43400, Selangor, Malaysia; hana_ridz@yahoo.com (R.R.); g_miah@yahoo.co.uk (G.M.); magajiusman0@yahoo.com (M.U.); 2 Department of Crop Science, Faculty of Agriculture, Universiti Putra Malaysia, Serdang 43400, Selangor, Malaysia; martinimy@upm.edu.my; 3 Department of Plant Protection, Faculty of Agriculture, Universiti Putra Malaysia, Serdang 43400, Selangor, Malaysia; izera@upm.edu.my

**Keywords:** chili anthracnose, *Colletotrichum*, breeding, marker-assisted selection

## Abstract

Chili anthracnose is one of the most devastating fungal diseases affecting the quality and yield production of chili. The aim of this review is to summarize the current knowledge concerning the chili anthracnose disease, as well as to explore the use of marker-assisted breeding programs aimed at improving anthracnose disease resistance in this species. This disease is caused by the *Colletotrichum* species complex, and there have been ongoing screening methods of chili pepper genotypes with resistance to anthracnose in the field, as well as in laboratories. Conventional breeding involves phenotypic selection in the field, and it is more time-consuming compared to molecular breeding. The use of marker-assisted selection (MAS) on the basis of inheritance, the segregation ratio of resistance to susceptibility, and the gene-controlling resistance may contribute to the development of an improved chili variety and speed up the selection process, while also reducing genetic drag in the segregating population. More importantly, by using molecular markers, the linkage groups are determined dominantly and co-dominantly, meaning that the implementation of a reliable method to produce resistant varieties is crucial in future breeding programs. This updated information will offer a supportive direction for chili breeders to develop an anthracnose-resistant chili variety.

## 1. Introduction

*Capsicum* is, at least economic-wise, one of the important vegetables worldwide. Global production of this vegetable in 2016 was approximately 34.5 million tonnes for fresh chili, and 3.92 million tonnes for dry chili [1]. *Capsicum* consists of herbaceous vegetables and spices grown in both tropical and subtropical regions and has approximately 30 well-known species [2,3]. This genus is assumed to have been selected in two areas of origin—the primary center and secondary centers [4]. Secondary centers can be described as the other regions which were introduced by the diversity of the capsicum, such as Brazil [5]. Among the chili-producing countries, including China, Indonesia, Mexico, Korea, Nigeria, Ghana, and Turkey, India stands as the largest producer with 33% of the world’s chili production [6]. Five species of *capsicum* (*C. annuum*, *C. baccatum*, *C. chinense*, *C. frutescens*, and *C. pubescens*) are commonly domesticated and cultivated in different parts of the world. But among these five species, *C. annuum* is the most commonly cultivated worldwide [7] followed by *C. frutescens* [8]. *Capsicum annuum* L. is a dicotyledonous flowering plant, grown on more than 1.5 million hectares of land worldwide [6]. Genetically, the *capsicum* species are diploid and can be distinguished into two species groups—one with 2*n* = 24, and another with 2*n* = 26 [2].

Globally, most chili production is affected either by biotic factors, such as phytopathogenic fungi, bacteria, viruses, and weeds; and other pests, including root-knot nematodes, aphids, and thrips; or abiotic factors, such as temperature, moisture, light, pesticides, and herbicides. Although several practices and precautions are implemented during chili planting, anthracnose is most likely to be the main constrictive factor in the postharvest stage. Anthracnose is a seed-borne disease that has caused a marketable yield loss of approximately 50% of the chili production in Malaysia [9], a 15% yield loss in Korea [10], an annual chili production loss of more than 35% in Indonesia [11], and an approximately 80% yield loss (caused by severe epidemics) in Thailand [12]. United States and Brazil are also facing this challenge [13,14]. This disease also affects the quality of fruits [15] by reducing the quantities of fruits’ dry weight, capsaicin, and oleoresin [16]. Anthracnose, caused by the *Colletotrichum* species, has been identified in many plants, and the symptoms appear on the its stems, leaves, flowers, and fruits [17]. The anthracnose disease caused by *C. truncatum* (synonym *C*. *capsici*) has been most commonly found in chili (*C. annuum* L.) [18] and has a wide host-range of more than 460 associated plant species [19], such as pumpkin (*Cucurbita moschata* (Duch.) Poiret) [20], papaya (*Chaenomeles speciosa* (Sweet) Nakai) [21], and tomato (*Solanum lycopersicum* L.) [22]. In the US, *C. acutatum* is considered to be the most destructive species of *Colletotrichum* as it affects both ripe and unripe pepper fruit, while *C. gloesporioides* only affects ripe pepper fruit [13].

Several strategies have been implemented to try and control anthracnose, such as the use of rotation crops, and application of the plant extracts as foliar sprays, like using the combination of neem (*Azadirachta indica*), mahogany (*Swietenia mahagoni*), and garlic (*Allium sativum*), which has been shown to have a significant impact in controlling chili anthracnose [23]. Chemical fungicides also help by applying quinone outside inhibitors (QoI), such as quadric and amistar [24,25,26] propiconazole (0.1%) treatment [27,28], mancozeb (0.2%) [29], and fludioxonil [30], as well as bio-fungicides, such as *Streptomyces* spp. [31]. Proper seed selection and the production of healthy seeds by applying 0.5–1.0% fungicide, such as a Thiram solution, is important for germinating the clean and disease-free seeds. Although fungicide sprays may be helpful, applications of these are only moderately effective under environmental conditions that are advantageous for pathogen infection. In some countries, such as Thailand and Indonesia, fungicides are considered uneconomical and not sustainable for small-holder farmers due to their high risks to environmental safety [18,32]. Thus, one of the most economical and significant strategies to reduce crop losses is to cultivate resistant varieties, or hybrids. The development of disease-resistant chili cultivar is an important requisite to overcoming the use of agrochemicals. 

Compared to conventional phenotypic screening, the application of molecular markers is a low-cost, high-throughput method for detecting disease-resistant genes, allowing the introgression of genes into susceptible varieties and the pyramiding of multiple genes into individual lines. However, the success of the breeding program in developing durable resistant varieties has been limited due to the association of multiple *Colletotrichum* species in anthracnose infection [33,34,35], along with the differential capabilities of the pathogenic virulence [18]. The use of resistant varieties not only eradicates anthracnose, but also removes the chemical and mechanical responses to the disease [36]. According to Azad [15], there is a positive correlation between the capsaicin content in *capsicum* and prevalence of the anthracnose disease. Normally, the capsaicin content is high in anthracnose-resistant varieties and low in susceptible varieties. Tenaya et al. [37] also agreed that a higher capsaicin level in red pepper (*C. annuum*) was associated with a greater resistance to anthracnose. The level of capsaicin content in chili can thus be used as a predictor for their susceptibility to the anthracnose disease, meaning that the determination of the level of anthracnose resistance not only provides information on a molecular level, but also about chemical content as well.

## 2. Chili Anthracnose

Anthracnose, which is derived from a Greek word meaning “coal,” is the common name for plant diseases with very dark, sunken lesions and containing spores from fungal species [38]. Anthracnose of chili is one of the most destructive and damaging fungal diseases in the pre-harvest and post-harvest stages in chili-growing areas, including tropical Asia [33,39,40,41]. This disease has too emerged as a major problem for ripened fruits, which is why it is also named “ripe fruit rot” [42]. Anthracnose on chili was first reported in New Jersey, USA, by Halsted [43] in 1890 who noted that *Gloeopsorium piperatum* and *Colletotrichum nigrum* were the causal agents. The taxa were later noted to be related to *C. gloeosporioides* by von Arx [44]. 

According to Mistry et al. [16], besides fruit rot, anthracnose also causes leaf spots, dieback on the stem, and seedling blight, or damping off. Typical symptoms of anthracnose on chili fruit include dark spots, sunken necrotic tissues, or water-soaked lesions, with concentric rings of acervuli. In some circumstances, the lesions are brown before turning black due to setae and sclerotia formation, especially when the infected area spreads rapidly due to excessive irrigation or rain on immature pods [45]. A small anthracnose lesion on chili fruits affects their market value, fruit weight, and their quantities of capsaicin and oleoresin [16,46,47], and the symptoms do not progress until the fruit ripens. 

Several important factors contribute to the occurrence of the anthracnose disease, including climate, seeds, and genetics. According to Rajapakse and Ranasinghe [48], the degree of anthracnose prevalence could vary depending on the seasonal conditions. An association with high relative humidity or rainfall frequency and high temperature with anthracnose epidemics on chili plants has been recognized, but relative humidity was found to be the most important climatic parameter related to anthracnose development in the chili [10]. The fungus is sustained in alternate hosts, infected seeds, and crop debris, and infection arises during times of excess irrigation or rain on immature pods. Conversely, the symptoms are not found until the pod becomes mature and completes its final color change.

## 3. *Colletotrichum* spp. and Occurrence

Anthracnose disease is caused by species of the genus *Colletotrichum*, which belongs to the kingdom fungi (*Ascomycota*). This genus was originally described as *Vermicularia* by Tode [49]. The *Colletotrichum* species are the most destructive plant fungi; they attack a variety of crop plants and cause damage to them, such as on the leaves, stems, roots, flowers, and fruits. Several species of *Colletotrichum* have been reported around the world as being the main cause of anthracnose in chili, including *Colletotrichum truncatum* (Sydow) Butl. and Bisby, *Colletotrichum gloeosporioides* (Pent.) Penz. and Sacc., *Colletotrichum acutatum* Simmonds, *Colletotrichum coccodes* (Wallr.) Hughes, and *Colletotrichum graminicola* (Ces.) Wils (Table 1)—this genus is listed as one of the top 10 fungal pathogen groups in molecular plant pathology [50]. Among these species, *C. truncatum* has been identified as the most common and dominant pathogen of chili anthracnose in some countries [9,51]. In addition to *C. truncatum*, *C. gloeosporioides* was also frequently isolated. Although both *C. acutatum* and *C. cocodes* were isolated also, their occurrences are very low in chili plants in Malaysia [9]. However, some countries reported that *C. acutatum* is the most critical among the anthracnose pathogens [13,46,52].

**Figure 1:**
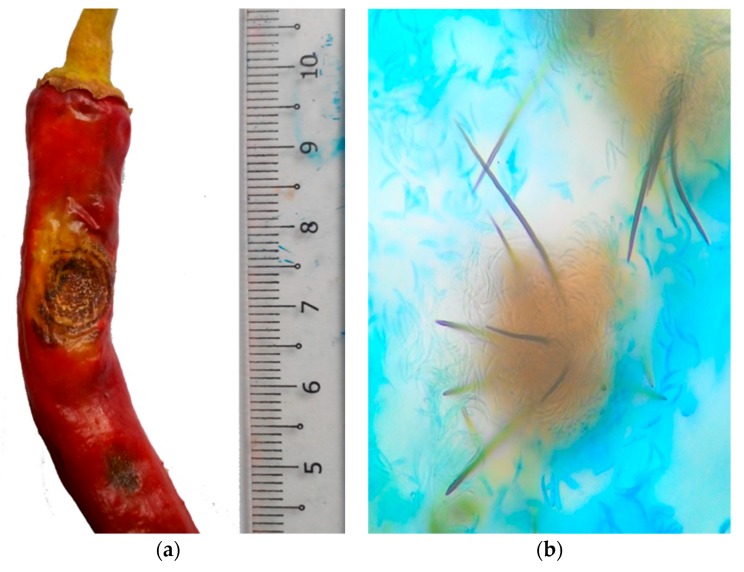

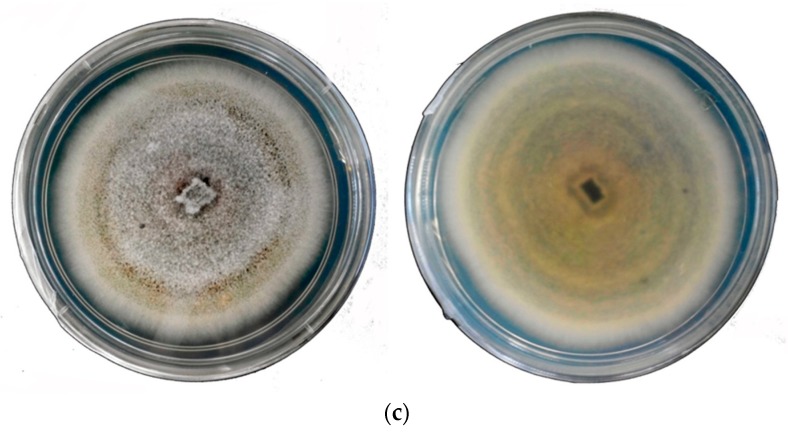
(**a**) Appearance of anthracnose on chili fruit: Dark and sunken necrotic tissues lesion with concentric rings of acervuli (measurement in cm); (**b**) presence of setae in acervulus (40×); (**c**) *Colletotrichum truncatum* (Syd.) E.J. Butler and Bisby.

The *Colletotrichum* species can be identified by their morphological or molecular characteristics. Some morphological characteristics, such as culture colony appearances, conidial morphology, growth rate, appressorial morphology, and the existence of septation, are important characteristics which help distinguish the *Colletotrichum* species. *C. truncatum*, the major pathogen of anthracnose, appears on potato dextrose media as a white to grey color with a dark green center, and a cottony mycelium with falcate conidia (Figure 1). The colonies produced by *C. gloeosporioides* are pale grey to black, zonated colonies, with ample orange conidial masses near the center—whereas *C. acutatum* is characterized as an orange-colored colony with slight mycelium [53].

## 4. Chemical Components in *Capsicum* and Their Relation to Anthracnose Disease

*Capsicum* belongs to the family *Solanaceae* and is native to Central and South America [70]. Chili can be described as a pungent variety of any *capsicum* species. Alternatively, “pepper” is a broad term to describe the fruits of any *capsicum* species with pungent (e.g., hot pepper) and non-pungent (e.g., sweet pepper) characteristics [71]. Chili has can be used in a multitude of ways—not only for its color, flavor, and spice, but also for its chemical, medicinal, and nutritional properties. Chili is also important for its color and pungency. The red color (oleoresin) of chili is mainly due to the presence of carotenoids, at a range of 0.3% to 0.8% in fruits [72]. According to Wesolowska et al. [73], capsanthin and capsorubin are the two main compounds responsible for the red color of chili (minimum 30% of the total carotenoids), while other colored compounds in chili fruit are zeaxanthin, cryptoxanthin, and lutein. The color extracts, known as paprika extract, contain a very low pungency and are broadly used in the food industry as a natural flavoring and coloring agent for many foods, such as cheese coatings, spicy foods, popcorn oil, meat products, and cheeses. 

On the other hand, the pungency or hot taste of *capsicum* is due to the presence of nonvolatile alkaloids—namely, capsaicinoids [74]. Capsaicinoids are biosynthesized and accumulated in the placenta of *capsicum* fruits, which is the part where seeds are usually attached [75,76,77]. The major capsaicinoid varieties are capsaicin (tran-8-methyl-*N*-vanillyl-6-nonenamide), present in most varieties of chili pepper, and dihydrocapsaicin (8-methyl-*N*-vanillylnonanamide), which is responsible for approximately 80–90% of the spiciness [78,79,80,81]. Minor capsaicinoids (homocapsaicin, nordihydrocapsaicin, norcapsaicin, nornorcapsaicin, nornornorcapsaicin, and nonivamide) are also found in chili peppers, but just to a lesser extent [82]. The length of the aliphatic side chain, the presence or absence of a double bond, the branching point, and their relative pungencies are the main differences between the capsaicinoids.

Capsaicinoids are important in food and pharmaceuticals due to their ability to stimulate the senses in the mouth and skin by providing a warm sensation [71]. Because they possess antioxidants [83], antimicrobial properties [84], and are anticarcinogenic [85], in appropriate doses they may increase energy metabolism, suppress fat accumulation [86], and offer anti-inflammatory effects [87]. However, the application of capsaicinoids is limited, because in higher doses they may cause irritation due to their pungency. A linear relationship between the total amount of capsaicinoids and pungency was reported by Krajewska and Powers [88] and Usman et al. [89]. The total pungency value of a specified sample was found by adding up the pungency values of the specific capsaicinoids. Thus, cultivars must have a specific pungency level to have commercial value. In addition, Saxena et al. [90] stated that fresh green chili fruits comprised of more vitamin C than that found in citrus fruits, while red chili fruits had more levels of vitamin A compared to that in carrots.

Walter [84] was the first researcher who suggested that the antimicrobial properties of capsaicin was a base component in the product that belongs to the group of biopesticides. According to Gudeva et al. [91], capsaicin is a naturally-occurring substance with no toxic effect, and which belongs to the third class of biopesticides. Therefore, it is suggested that this antimicrobial capacity of capsaicin, or any related compounds of phenol groups, may contribute to their anthracnose resistance. Ko et al. [92] found that ripe pepper fruits (*Capsicum annuum*) were resistant to *Colletotrichum gloeosporioides*, while unripe, mature fruits were more susceptible. Similarly, Oh et al. [93] and Kim et al. [94] observed a greater prevalence of appressorium and infection hypha development on unripe fruit compared to ripe fruit. 

Some researchers emphasized that physiological changes (changes in pH and cell wall composition, decrease in fruit firmness, and increased soluble sugars and secondary metabolites) during the ripening stage could increase the chili pepper’s susceptibility to *Colletotrichum* spp. infection [95,96,97,98]. However, this statement contrasts with the report mentioned previously, which stated that ripe fruit were more resistant to *Colletotrichum* spp. A study by Susheela [59] also stated that the pathogenicity of *Colletotrichum* spp. was higher during the chili’s ripening stage, as compared to unripe period. However, it was found that the infection generally depended on the species or varieties of chili pepper, or the *Colletotrichum* species tested. 

Prasath and Ponnuswani [99] conveyed that phenol and enzymes helped to resist formation of fungal infection on the chili pepper. Two polyphenols in the fruits of *C. annuum*, *N*-caffeoyl putrescine and caffeoyl *O*-hexoside, seemed to act as a phytoalexin in the defence mechanism against *C. gloeosporioides*. Other polyphenols—feruloyl *O*-glucoside, kaempferol *O*-pentosyldihexoside, and dihydroxyflavone *O*-hexoside—were constitutively formed as a phytoanticipin in the infected *C. annuum* fruits [100]. Phytoalexins are the secondary metabolites produced de novo by plants in response to biotic and abiotic stresses, whereas phytoanticipins are the constitutive metabolites with a defensive role [101]. Host resistance in these species is differentially expressed at the different stages in fruit maturity [18,102].

## 5. Screening Method for Chili Anthracnose

The most common methods used to inoculate *Colletotrichum* on chili plants either involve using the fruit puncture method or the spraying method in the laboratory or in the field [59]. The fruit puncture method involves the injection of a very small amount of conidia suspension into the fruit pericarp, which is also known as the microinjection method. The resistant varieties are successfully determined according to the lesion formed at the injection or inoculation area [54,102,103]. Alternatively, the spraying method does not involve any wounding, as the conidia suspension is only sprayed on the plant during the flowering and fruiting stage. However, the spraying method is considered unsafe and less accurate due to its risks to the environment. The microinjection of detached fruits in the laboratory has contributed to the excellent progress of anthracnose resistance evaluation in chili fruits. Currently, less emphasis has been employed on field assessments of candidate lines and selections [46]. 

Several implementations have been done to assess anthracnose diseases, including the spraying method and microinjection method. Rajapakse and Ranasinghe [48] implemented the pin prick method in the laboratory to test the ability of *C. truncatum* isolates to produce anthracnose lesions on the fruits. They then used the spraying method in the field, and found that the spraying method displayed a more effective result for screening the chili varieties during the red stage. Montri [18] conducted a pathogenicity study of *C. truncatum* using the injection method on the red-ripe stage of chili. A total of three pathotypes were identified from 11 isolates of *C. truncatum* collected, based on the aggressiveness of the isolates. Research by Mahasuk et al. [104] reported that two different inoculation methods had been implemented for anthracnose disease screening: the microinjection (MI) method, and the high pressure (HP) spraying method. Similarly, for Susheela [59], two methods were tested to evaluate anthracnose, applying the fruit puncture method in the laboratory and the spray inoculation method in the field. The hybrids were rated phenotypically as either resistant or susceptible, based on the anthracnose lesion area (mm) and disease incidence (%). 

Overall, both the lab and field methods indicated a positive correlation in disease occurrences during the red stage. However, under laboratory conditions, the fungus seems to penetrate more in the green stage compared to the red stage. In contrast, the spraying method indicated high disease incidence during the red stage compared to the green stage. This finding indicated that the conditions of the plants/fruit should be considered both in nature or in the field, as well as in the lab. Susheela [59] emphasized that by wounding the fruits during the laboratory study, the fruits developed sclerenchyma-like cells, which led to the formation of an anthracnose lesion. 

As mentioned previously, anthracnose resulted in fruit yield loss and reduced fruit quality in both green and red chili fruit. Among three important *Colletotrichum* spp. (*C. truncatum*, *C. gloeosporioides*, and *C. acutatum*), *C. acutatum* is the most virulent species, attacking both green and red fruit, followed by *C. truncatum* and *C. gloeosporioides* [34]. *C. truncatum* infected more red fruits [53] whereas *C. acutatum* and *C. gloeosporioides* occurred on both young and mature green fruits [13]. In regard to the leaf part, *C. cocodes* infected more young leaves than mature leaves [105]. Commonly, anthracnose is found on mature red fruits and leads to both pre- and post-harvest fruit decay [8]. Many post-harvest fruit diseases display the quiescence phenomenon, where symptoms do not progress until the fruit ripens. Rajapakse and Ranasinghe [48] found that chili fruits at a ripe red stage were more susceptible to *C. acutatum* than green fruits, in that the lesion diameters of anthracnose on green fruits were smaller compared to the lesion diameters on ripe red fruits at seven days after inoculation.

## 6. Markers Available for Disease Resistance

Ansari [106] divided disease resistance into two modes of inheritance: monogenic disease resistance, or polygenic disease resistance. Monogenic disease resistance involves a single gene that is either dominant or recessive, which is responsible for disease resistance. Unlike polygenic disease resistance, monogenic disease resistance is clearly defined, and the gene, either dominant or recessive, can be determined. The possibility of identifying a linked marker to the disease resistance gene is contrariwise proportional to the genetic distance between the gene and the marker. For an accurate estimation, the genetic distance between the gene and the marker should be calculated from a bulky population or from several crosses. For this reason, the genetic distances may differ significantly between crosses [107]. Linkages have usually been detected between markers and monogenic disease resistance by constructing a genetic linkage map. 

**Figure 2:**
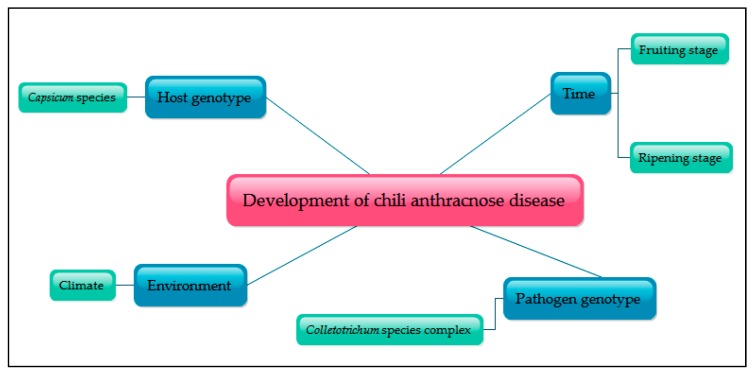
Four main factors in chili anthracnose disease development.

Polygenic resistance disease is conferred by several genes (more than one) and can be difficult to identify individually. Almost all quantitative (complex) disease resistances are under oligogenic or polygenic control [108] and are influenced by environmental factors. This problem can be solved by identifying chromosome sites through quantitative trait loci (QTLs) [109]. The quantitative resistance against certain biotic and abiotic stresses may lead to the simultaneous and independent allelic variation of such genes, influenced by the effect of the environment [110,111]. The following four factors may be responsible for the development of anthracnose disease: host genotype, pathogen genotype, time, and the environment (Figure 2).

Generally, three different types of markers—morphological, biochemical, and molecular—are used for distinguishing components in plants. Out of these three, molecular markers precisely and directly reveal DNA polymorphism [106]. Molecular markers are different from morphological markers in terms of dominant and codominant character, meaning that both parental markers can be observed in the progeny. Molecular markers define a fragment of DNA that differs in nucleotide sequences and is unaffected by growth stage, agronomic practice, season, or location [112]. In marker-assisted selection, the application of molecular markers is not affected by the environment, does not interact with other genes, and significantly simplifies the inheritance pattern for complex traits. 

Various molecular markers such as random amplified polymorphic DNA (RAPD), inter-simple sequence repeat (ISSR), simple sequence repeat (SSR), restriction fragment length polymorphism (RFLP) and amplified fragment length polymorphism (AFLP) are used for variability and diversity assessments at the molecular stage. Kang et al. [113] successfully constructed a molecular linkage map of the chili pepper (*capsicum* spp.), with RFLP and AFLP markers being particularly related to carotenoids and capsaicinoids. For example, the red color of *capsicum* is determined by a single dominant gene. However, the use of radioactivity and its labor-intensive nature are limitations of RFLP markers in molecular research [114,115]. The combination of molecular diagnostic tools and the use of different isolates could be an appropriate and dependable tactic for learning about the pathological variability in the *Colletotrichum* species. Molecular marker methods have been effectively employed in the past to detect variations among the anthracnose pathogens (*C. truncatum*, *C. gloeosporioides*, *C. acutatum*, *C. coccodes*, *C. dematium*, and *Glomerella cingulata*) using the random amplified polymorphic DNA polymerase chain reaction (RAPD-PCR) technique [33,116]. 

In contrast, SSR markers are highly variable, reproducible (when using the polymerase chain reaction (PCR)), co-dominant, multiallelic types of variations [117,118]. The DNA fragment is short, with less than 6 base pair repeated motifs [119]. SSRs are also known as microsatellites [120] and short tandem repeats (STRs) [121], and produce more information compared to other markers due to their uniformity and abundance in eukaryotic genomes. SSRs are applied in genome mapping, gene tagging, the estimation of genetic diversity, variety identification, quantitative trait loci (QTL) analysis, and MAS [122,123,124]. Many genetic mapping and diversity studies of the chili have been reported in the past [121,125,126]. According to Geleta et al. [127], the effective prediction of heterosis and performance of F_1_ hybrids has been reported in SSR markers based on the morphological similarity with their parent plants.

There are some limitations when using molecular markers, summarized as follows:

- All markers are not breeder-friendly. This difficulty can be minimized by altering non-breeder-friendly markers to new types of breeder-friendly markers e.g., RAPD to sequence characterized amplified region (SCAR) and RFLP to sequence-tagged sites (STS).
- All markers are not appropriate for whole populations, due to the absence of marker polymorphisms or trustworthy marker-trait association. Multiple mapping populations are used to better identify genetic background effects and marker allelic diversity.
- Incorrect selection may occur due to recombination between the markers and the genes/QTLs of interest. In that case, using flanking markers or many markers for the target gene/QTL can help achieve consistent selection [128].
- Incorrect estimations of QTL locations and effects may result in delayed advancement. High marker density with fine mapping and well-designed phenotyping across various environments and large populations may deliver more precise estimates of QTL locations and effects. The efficiency of QTL detection depends on algorithms, the number of polymorphic markers, mapping methods, and the population type and size [129].
- The marker-assisted backcrossing (MAB) methods and schemes are comprehensible, suitable, and implementable for plant breeders, unless designed for large-scale use in practical breeding programs.
- A huge quantity of breeding programs have not been furnished with satisfactory facilities and situations for a large-scale implementation of MAB in practice.
- In many cases, laboratory expenditures and labor charges are still considerable [130].

Next generation sequencing (NGS) is the latest approach that provides plant breeders a powerful tool for the development of superior cultivars. The ability of NGS to detect large numbers of DNA markers within a short time period has reduced the cost of whole genome sequencing (WGS) by many folds, allowing for the discovery, sequencing, and genotyping of thousands of markers in a single step [131]. This technology has been applied for marker discovery and targeted resequencing to identify domestication-related genes by comparing the genomes of crop species and their wild relatives [132], as well as for genome-wide selection studies to predict the breeding value of traits [133]. NGS technologies have identified the anthracnose resistance gene in lupin [134], walnut [135], and alfalfa [136]. Kim et al. [137] sequenced and assembled *C. annuum* cv. CM334 (Criollo de Morelos 334) from a Mexican state with a genomic size of 3.48 Gb, which can be used extensively in improving disease resistance of the *capsicum* species.

## 7. Conventional Breeding to Marker-Assisted Breeding for Anthracnose Disease Resistance

**Figure 3:**
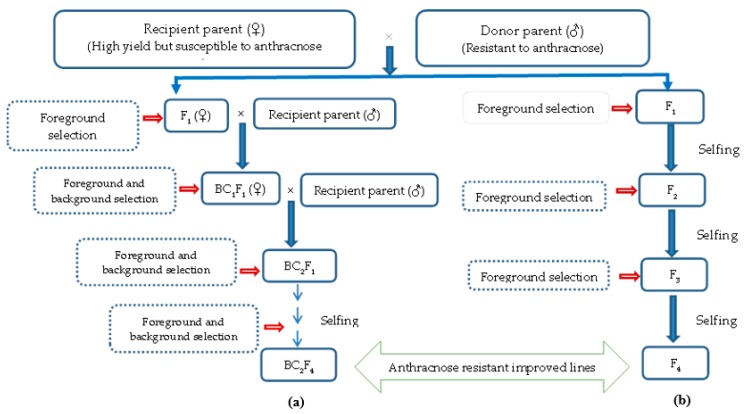
Diagram showing the development of anthracnose-resistant varieties through (**a**) marker-assisted backcrossing and (**b**) pedigree selection.

Breeding involves hybridization between contrasting parental lines and the selection and evaluation of hybrids before testing and releasing new varieties [138]. Conventionally, although the phenotypic selection of hybrids was conducted by planting in a glasshouse or field trials, this method is quite time-consuming. Some methods commonly used in conventional breeding are the pedigree, backcrossing, and recurrent selection methods [138]. Crossbreeding and the selection of elite lines in succeeding generations would develop the ideal chili genotypes [139]. Pedigree breeding is the most common method used to specially develop crop plants with resistance to insects and diseases, if the traits are governed by major genes and have higher heritability. For those traits with low heritability, the selections are often delayed until the lines become more homozygous in later generations (F_5_ or F_6_) [140]. Voorrips et al. [55] implemented a quantitative trait locus (QTL) mapping approach to study the inheritance of anthracnose resistance in an F_2_ population derived from the pedigree of *C. annuum* × *C. chinense*. Backcrossing is one of the most important techniques used in chili breeding for the introgression of a target gene from a donor parent to a recipient parent. Successful resistant lines have been reported to be derived from the backcrossing breeding of a susceptible chili pepper genotype, *C. annuum*, with resistant chili pepper genotypes, *C. baccatum* and *C. chinense* [141,142,143,144]. This technique also involves diallel crosses, which involves two desired traits. According to Johnson [145], diallel crosses are systematic, analytical, and comprehensive genetic evaluations, beneficial for recognizing the best selection potential for crossing at initial generations. Recurrent selection is another traditional breeding method used for male sterility which permits shorter breeding cycles, a more precise follow-up of genetic gains, and which provides opportunities to develop wide-range genetic diversity breeding lines [146]. The general methodology of producing an anthracnose disease-resistant variety by using backcross and pedigree selection is shown in Figure 3.

Marker-assisted selection is a significant tool for the breeding of chili and is implemented to select progenies that already have a scored trait. Specifically, breeders apply marker-assisted selection to an important trait that is difficult to measure and use a particular electrophoretic band that has been shown to be tightly linked to the desired trait using molecular/DNA markers. These markers are vital tools for improving the competence of the MAS process and permit for the identification of individual plants that have a greater recurrent genome percentage, and also assist in the selection of plants that convey a target marker [147,148]. In the chili breeding program, MAS offers the most economical, fast, accurate, reproducible, and environmentally-friendly method to develop superior chili varieties with a certain degree of disease tolerance. In developing disease-resistant varieties, the susceptible and resistant genotypes to the anthracnose disease were precisely identified using the molecular marker at an early stage of plant growth, compared to field screening with artificial inoculation, without any environmental influence. 

Alternatively, some terms are used to describe several modern breeding strategies, including marker-assisted selection (MAS), marker-assisted backcrossing (MABC), marker-assisted recurrent selection (MARS), marker-assisted pedigree selection (MAPS), and genome-wide selection (GWS) or genomic selection (GS) [149,150]. Marker-assisted backcrossing (MABC), as a type of MAS, is a superior and effective method to introduce genes of interest while retaining the essential characteristics of the recurrent parent. Most of the important traits are quantitatively inherited. This method has been employed by many breeders because it is cheaper and faster compared to the conventional phenotypic selection method. Moreover, the presence of the recessive allele also can be precisely determined, and it is also unaffected by environmental conditions [151]. 

Recently, the introduction of new cultivar as a source of valuable yield and quality genes has been demonstrated in chili. These studies presented evidence that new varieties could contain favorable gene potential that can be introduced into the present cultivar. The use of host plant resistance is one of the most imperative mechanisms of the breeding and disease-controlling approaches. Moreover, the development of resistant varieties is important as the first step to fighting against infestation of insects/pests, and diseases with an implemented eco-friendly disease management program may reduce the use of chemical pesticides, which can assist in the organic cultivation of chili crops. Breeding for anthracnose resistance was started in the early 1990s [152,153], involving some *capsicum* species with a potential resistance trait, such as *C. annuum*, *C. frutescens*, and *C. baccatum*. Park et al. [154] found resistant features in *Capsicum baccatum* lines and suggested that, compared to other *capsicum* spp., the *C. baccatum* germplasm pool contained higher levels of resistance to anthracnose, which may prove useful as genetic resources for anthracnose resistance. The molecular markers reported associated with anthracnose-resistant traits in *capsicum* (MAS) is presented in Table 2.

Concerning *C. annuum*, no commercial resistant varieties have been developed, due to the lack of resistance in the *C. annuum* gene pool [157]. The introgression of the resistance gene from *C. baccatum* to *C. annuum* is difficult. For example, PBC80 was introduced into *C. annuum* via a trispecies cross by using *C. chinense* as a bridge [158]. There are several studies focusing on the introgression of anthracnose resistance into *C. annuum* to develop a new variety [159,160]. Hasyim et al. [46] listed five lines of *C. annuum* from AVRDC, Taiwan, namely AVPP1102-B, AVPP0513, AVPP0719, AVPP0207, and AVPP1004-B, as the promising lines with good fruit yield and tolerance to anthracnose. Two chili varieties from IVEGRI, Indonesia, Lembang-1 and Tanjung-2, have been reported as moderately resistant to anthracnose [161]. In India, some anthracnose-resistant lines listed by Reddy et al. [162] are Bhut Jolokia [163]; LLS, PBC932 (VI047018), Breck-2, PBC80 (VI046804), Breck-1, Jaun, and PBC81 (VI046805) [164]. New crosses were made to combine superior sources of disease resistance, such as PBC932, a *Capsicum chinense* germplasm selection, with elite Indonesian OP varieties, mainly “Jatilaba”, “TitSuper”, and “KR-B” (“Keriting” from Bogor). Thus, the identification of genetically superior parents is an important prerequisite for the development of elite strains, meaning that varietal identification and differentiation should be obtained with reproducible genetic information. 

The application of MAS is crucial in the chili crop improvement program, especially for the efficient selection of many resistance genes that pyramid into a single genotype. The interspecific cross between “Yeoju” (susceptible) and ‘Daepoong-cho’ (resistant), a local Korean variety of *C. annuum*, demonstrated that anthracnose resistance against *C. truncatum* is controlled by a monogenic recessive gene [165]. In contrast, the recent genetic analysis of F_1_ and BC_1_ of *C. chinense* (PBC932) by Sun et al. [141] indicated that the resistance of PBC932 to *C. acutatum* is mostly dominant, and was observed in chromosome P5 for both green and red fruit. Similar experiments conducted by Lin et al. [166] demonstrated that the resistance of PBC932 against *C. acutatum* was controlled by two dominant genes at the green fruit stages and two recessive genes at the ripe fruit stage. Research on the *C. annuum* breeding line ‘83-168’ by Lin et al. [167] found that the resistant character towards *C. truncatum* is inherited by a single dominant gene. Research by Mahasuk et al. [168] indicated that the resistance genes of *C. baccatum* PBC80 are controlled by single dominant genes. Previous research by Yoon and Park [158] also indicated that a dominant gene was the resistance gene of *C. baccatum* ‘PBC80’ to an isolate of *C. acutatum* KSCa-1. However, the resistance genes in *C. chinense* and *C. baccatum* were differentially expressed at different fruit maturity stages. Alternatively, some recent research reported that the inheritance of anthracnose resistance is controlled by recessive genes. In mature green fruit, the resistance gene is the recessive gene *co1* [168], while in ripe fruit and seedlings, the recessive genes *co2* and *co3*, respectively, are responsible for anthracnose resistance [144]. Mahasuk et al. [168] found that the resistance at the ripe red fruit and mature green stages is controlled by a single dominant and single recessive gene, respectively, between an intraspecific cross derived from *C. baccatum* PBC1422 and PBC80. Suwor et al. [142] phenotypically and genotypically demonstrated the resistance of AVPP0207 (*C. annuum* progressive line derived from PBC932) against two isolates of *Colletotrichum acutatum* and *C. truncatum*. He found that the resistance gene (AVPP0207) was located on chromosome P5 and thought it might be conditioned by the minor genes of the susceptible parent. Ko et al. [92] reported that differential resistance in the green and ripe fruit of the same chili variety may be due to high expression of the *PepEST* gene. 

The interactions between the chili pepper (as host) and the pathogenic *Colletotrichum* was first explained by a gene-for-gene relationship proposed by Flor [169]. The expression of resistance or susceptibility of the host to a specific pathogen is restricted to the pathogen genotype, and the degree of pathogen virulence found on the host genotype. The corresponding gene pairs regulate the outcome of any particular genotype-genotype interaction. In chili pepper breeding, Lee et al. [155] emphasized the importance of codominant marker development because it is difficult to distinguish between homozygous and heterozygous resistant plants using the phenotype. Based on his finding, anthracnose resistance is controlled by a major resistance locus. Other researchers also stated similar and contrasting findings, such as resistance to *C. truncatum* being inherited recessively with significant epistatic interactions using generation mean analysis [170]. Ahmed et al. [171] found that the resistance was controlled by polygenes with a predominantly additive type of gene action, while Qing-Lin et al. [172] found evidence for a monogenic dominant inheritance of resistance to *C. truncatum.* Single marker analysis by Nanda et al. [173] and Neetha et al. [174] found that markers HpmsE 081 and HpmsE 047 were associated with genomic regions controlling anthracnose resistance. The variation of the inheritance pattern in anthracnose resistance seems to cause significant constraints in the conventional breeding of chili. Thus, an attempt ought to be made to understand the dynamics of host-pathogen interaction. 

## 8. Present Progress and Outlook

Following the recent endeavors of plant breeders to improve on and produce disease-resistant chili varieties, numerous traditional and modern breeding methods have been applied. The controlled crosses between individual plants produce a desirable genetic difference to be recombined and transferred to the next progeny through a natural process. MAS is an effective tool for the introgression of resistant genes into new plants and the efficient selection of many resistant genes for pyramiding into a single genotype. Understanding the mechanism of monogenic and polygenic disease resistance, as well as host-pathogen interaction, is crucial. Therefore, a study using PCR-based markers will be useful for breeding cultivars with enhanced resistance to anthracnose, for pyramiding resistance to *Colletotrichum* spp., and for the further characterization of the locus, including the isolation of genes responsible for resistance to anthracnose fruit rot.

## Figures and Tables

**Table 1:** The *Colletotrichum* Species, Reported as Being Causal Agents of the Chili Anthracnose Disease.

Region/Country	Pathogen	Source
Malaysia	*C. truncatum*	Sariah [9]; Mahmodi et al. [54]
Thailand	*C. acutatum*, *C. truncatum* and *C. gloeosporioides*	Than et al. [53]; Montri [18]
Indonesia	*C. acutatum*, *C. truncatum* and *C. gloeosporioides*	Voorrips et al. [55]
India	*C. truncatum*, *C. dematium*, *C. gloeosporioides*, *C. graminicola*, *C. acutatum*, *C. piperatum*, *C. atramentaum*, *C. truncatum*, *C. fructicola* and *C. siamense*	Thind and Jhooty [56]; Hedge et al. [57]; Paul and Behl [58]; Susheela [59]; Selvakumar [60]; Kaur and Singh [61]; Ramachandran and Rathnamma [62]; Sharma et al. [33]; Sharma and Shenoy [63]
Korea	*C. acutatum*, *C. gloeosporioides*, *C. coccodes* and *C. dematium*	Park and Kim [64]
Papua New Guinea	*C. truncatum* and *C. gloeosporioides*	Pearson et al. [65]
New Zealand	*C. coccodes*	Johnston and Jones [66]
Taiwan	*C. acutatum*, *C. truncatum* and *C. gloeosporioides*	Manandhar et al. [47]
Australia	*C. acutatum*, *C. atramentarium*, *C. dematium*, *C. gloeosporioides var. minor* and *C. gloeosporioides var. gloeosporioides*	Simmonds [67]
United Kingdom	*C. acutatum* and *Glomerella cingulata*	Adikaram et al. [68]
USA	*C. gloeosporioides*, *C. acutatum*	Harp et al. [13]; Roberts et al. [45]
Vietnam	*C. acutatum*, *C. truncatum*, *C. gloeosporioides* and *C. nigrum*	Don et al. [69]
Sri Lanka	*C. truncatum*	Rajapakse and Ranasinghe [48]

**Table 2:** Molecular Markers Associated with Anthracnose-Resistant Traits in Capsicum, with Marker-Assisted Selection (MAS).

*Capsicum* Species	*Colletotrichum* Species	Marker Type	Marker Name	Reference
*C. annuum* × *C. chinense* *C. annuum* × *C. baccatum*	*C. truncatum* *C. acutatum*	SSR, SCAR	HpmsE032, HpmsE143, InDel	Lee et al. [155]; Wang [156]; Suwor et al. [142]
*C. annuum* × *C. chinense*	*C. acutatum*	SSR, SCAR, CAPS	HpmsE057, HpmsE116, HpmsE126, ES382, Gpms161, Gp20068, ES64, Epms745, Gp20068, ES118, ES181, InDel, C2_At4g03400	Sun et al. [141]
*C. annuum* × *C. baccatum*	*C. truncatum* *C. acutatum*	SSR, CAPS	HpmsE032, HpmsE143, CaR12.2M1, CcR9M1	Lee et al. [155]
*C. annuum* × *C. baccatum*	*C. truncatum* *C. acutatum*	SSR	Hpms2-24, HpmsE143, HpmsE092, HpmsE032, HpmsE063	Lee et al. [143]
*C. annuum* × *C. chinense*	*C. gloeosporioides* *C. truncatum*	AFLP, SSR	B1, B2, D1, G1, H1, CA-MS6, CA-MS12, CA-MS22, CA-MS23, CA-MS25	Voorrips et al. [55]

Note. SSR: simple sequence repeat; SCAR: sequence characterized amplified region; CAPS: cleaved amplified polymorphic sequence; AFLP: amplified fragment length polymorphism.
